# Hemicrania continua- Major Shortcomings in the new classification or rigid thinking is death to progress

**DOI:** 10.1186/1129-2377-15-53

**Published:** 2014-08-28

**Authors:** Peter J Goadsby

**Affiliations:** 1 NIHR-Wellcome Trust Clinical Research Facility, King’s College London, London, UK

## 

Asked to write upon the matters arising [1] from comments [2] on an earlier contribution [3] is an unusual position that has allowed debate around the beta version of the third edition of the International Classification of Headache Disorders, specifically its definition of hemicrania continua [4]. The issue at hand is the extent to which further experience and clinical science should allow the first description of a syndrome to evolve over time. I am sorry if my nuanced title left one’s correspondents of a mind that I was expressing more than a general principle.

Regarding our development of a placebo-controlled indomethacin test for hemicrania continua [5], the purpose of the evolution was to introduce the scientific discipline of a control arm. It is generally accepted that the use of placebo can be traced to widely available texts of a slightly older vintage [6], over which priority in many ways would be jejune at best. The authors state “indomethacin is potentially a harmful drug”, a splendid justification for careful placebo-controlled assessment of the patient before longer term medicine exposure. Furthermore, the authors argue for an absolute limit on daily use of indomethacin at 200 mg per day. One recommends your correspondents consider the concept of a normal distribution, which predicts a rigid upper limit will fail to capture some cases. Now if there was a biological basis for such a rule this would be wonderful, however, since there is not, the dated, perhaps mid-twentieth century concept that “it is because we say so…” might usefully transform to a more objective advisory evolved through clinical science. Perhaps not recognized by your correspondents, the placebo-controlled indomethacin test is simply a way to bring order to study of the problem. We saw no harm in our series, and would submit labeling placebo control as not rational is, at best, a very curious use of the term.

On the subject of the cranial autonomic features, I am not sure why seven features as we have reported [5] constitute a “heap”, which I think is probably not the collective noun for a list of cranial autonomic features, when six, as the authors identified [7], is untouchably canonical? Moreover, by taking a very careful history, identifying the symptoms is perhaps easier if one is aware of the literature as it stood. The frequency difference seems utterly predictable by the passage of time and experience of enquiry. Your correspondents identify a human factor in the varying frequency of symptoms; one might christen that as bogus, although one might also conclude a less dogmatic, open scientific approach, as we have applied, is likely to see more than a less flexible form of rigid enquiry. The authors refer to the added cranial autonomic features being obligatory; the phrase- *at least one*[4] does not make anything obligatory.

Your correspondents seems content with the concept of adding features [8]; the essential issue seems to be what defines the “frame”. I would suggest data derived substantially from a placebo-controlled study should take precedence over opinion, no matter how exalted the source.

Regarding non-indomethacin unilateral headache, we have certainly seen this. I am sorry if I was not clear when I said “non is not an option” referring to an indomethacin response in hemicrania continua. What I should have spelt out is that for ICHD-III-beta [4], if there is no indomethacin response, the diagnosis is not made. I was not commenting upon the acronym itself. Unilateral headache without an indomethacin effect deserves careful study; this will require use of placebo, careful phenotyping and an open mind- acronym creation may serve those masters when the subject is better understood.

Your correspondents’ comment that the remark- *I would submit the diagnosis has not changed*- is meaningless and uninformative. The statement has a clear meaning, or the authors would not have commented to the meaning, and as a submission, it expresses an opinion; the correspondents seem both to have understood and been informed, if not pleased.

Lastly, your correspondents state the female Case One as described [9] would not have hemicrania continua by ICHD-III-beta. That patient has unilateral headache (A), it was present for more than three months with fluctuations (B). She “sometimes lay down in bed quietly” (C-2- by behavior). They report “indomethacin completely abolished her headache” (D), and she has no findings of abnormal neurology on examination or investigation, nor does she have another diagnosis to explain her problem (E). The patient described in 1984 had hemicrania continua [9], application of ICHD-III-beta [4] diagnoses hemicrania continua. One invites the correspondents to re-read ICHD-III-beta and criticise what is wrong, rather than bloviate by less than attentive application of the criteria.

## Competing interests

The author declares that he has no competing interests.
